# Is It Time to (Re)define the N‐Category for Metastatic Lymph Nodes in Non–Small Cell Lung Cancer?

**DOI:** 10.1111/crj.70016

**Published:** 2024-09-25

**Authors:** Koen C. H. A. Verkoulen, Jean H. T. Daemen, Aimée J. P. M. Franssen, Juliette H. R. J. Degens, Karel W. E. Hulsewé, Yvonne L. J. Vissers, Erik R. de Loos

**Affiliations:** ^1^ Department of Surgery, Division of General Thoracic Surgery Zuyderland Medical Center Heerlen The Netherlands; ^2^ Department of Pulmonary Medicine Zuyderland Medical Center Heerlen The Netherlands

**Keywords:** NSCLC, lymph node metastasis, TNM classification

In a recent issue of *The Clinical Respiratory Journal*, Guo et al. published a study that evaluated the correlation between the rate and number of resected metastatic lymph nodes and survival in patients undergoing an anatomical resection for non–small cell lung cancer (NSCLC) [1]. To date, nodal staging is key in the work‐up and treatment guidance for NSCLC as it is an important determinant of survival [2, 3]. As opposed to some other cancer types, nodal staging for lung cancer is based on the anatomic location of the respective regional and mediastinal lymph node stations rather than the number of metastasis [4, 5, 6]. Hence, the ongoing debate concerning the potential prognostic value of the number and rate of lymph node metastases in NSCLC continues. Over the last decade, numerous studies have aimed to address this issue [7, 8, 9, 10]. However, they are generally limited by their retrospective design and inherent bias, as well as methodological disparity [11]. How does the current report add to the evidence collected for over more than a decade?

Guo et al. carried out the first population‐based study concerning this subject. They revealed that both the number and rate of positive lymph nodes after lymphadenectomy concomitant to an anatomical lung parenchyma resection are a predictor for overall survival, independent of the anatomical location of the nodal station that is affected, being either N1 or N2. These results are in line with prior retrospective studies and a recently published meta‐analysis [8, 10, 12]. However, the number and rate of metastatic lymph nodes was only examined in postoperative patients that underwent lymphadenectomy, in whom the lymph nodes were completely dissected (defined as examination of more than 15 dissected lymph nodes) instead of biopsied stations. Thus, these results are only applicable as a prognostic tool and in treatment decision‐making processes for postoperative patients. To be of an even greater importance for treatment plan composition, for example, one should repeat this study for preoperative clinical lymph node staging (cTNM) using minimally invasive staging techniques like endosonographic lymph node staging (EUS/EBUS) or surgical video‐assisted mediastinoscopy (VAM) or video‐assisted mediastinoscopic lymphadenectomy (VAMLA). However, a recent publication showed in a noninferiority study that VAMLA might not be of added value in patients that underwent systemic EUS/EBUS [13]. Additionally, VAM/VAMLA or EUS is mainly used to assess N2 nodes. Hence, the vast majority of metastatic N1 nodes cannot be evaluated through these techniques. These drawbacks illustrate the challenges of the current TNM classification, and lymph node staging, especially for clinical lymph node staging. The sensitivity of preoperative lymph node staging modalities like (PET)‐CT scan and EUS/EBUS ranges from 20% to 70%, resulting in a relatively high rate of unforeseen N2 disease [14]. The sensitivity of invasive mediastinal staging like VAM/VAMLA ranges from 64% to 90%, which is higher, but is still prone to the fact that mainly only N2 nodes can be assessed [15, 16]. So, the method used by Guo et al. might not be applicable to clinical N staging (cN), but the current preoperative staging modalities are far from perfect either. This makes it difficult to redefine the cN‐category of the cTNM classification according to rate and number of metastatic lymph nodes, regardless of their location.

Approaches to redefine the N‐category of the TNM staging system have been proposed in the past. Examples are the nodal zone concept, single‐ or multi‐station N1 and N2 disease, and the exact location of N1 or N2 metastatic nodes, in which more proximal affected nodes (closer to the mediastinum) connote worse prognosis [11, 17]. The type of N2 disease, that is, single level, multilevel, or bulky disease, has also been associated with survival outcomes and could be used to redefine the N‐category [18]. However, these approaches for defining the N‐category mostly only apply to postoperative patients like in the study of Guo et al. or are subject to the same drawbacks of targeted biopsies and VAM/VAMLA, which do not provide the opportunity to assess the total number and rate of metastatic lymph nodes, especially in N1 nodes. Hence, some even question whether a change of the N‐category would even be for the better. In the end, the purpose of changing the N classification of the TNM classification should be about being able to better predict survival outcomes and, in turn, tailor treatment plans in a more patient‐specific approach. A good example of the path to more patient‐tailored treatment approaches is molecular testing. A technique that is used to detect gene mutations that play an important role in targeted therapy [19].

So, if we do want to continue the pursuit of improving the accuracy of nodal staging, more uniformity and clarity of the current guidelines is to be obtained. Disparities can be found in the European and American guidelines for preoperative lymph node staging. The revised European Society of Thoracic Surgeons guidelines for mediastinal staging recommend dissection of at least nodes 4L‐R and 7 [20], whereas the American College of Chest Physicians guideline recommends dissection of at least 2L‐R, 4L‐R, and 7 [21]. In several national guidelines, including the Dutch guidelines, specific stations that should be dissected are not even specified [22].

Similar disparities in recommendations apply to intraoperative nodal staging. Guo et al. recommend dissection of at least 16 examined lymph nodes and excluded patients with < 16 examined lymph nodes. A different study, however, recommended > 10 examined lymph nodes instead of the 16 proposed by Guo et al. [7]. Another study found that survival improves increasingly when more lymph nodes are dissected during anatomical lung resection [23]. On the contrary, the benefit of lymph node dissection over sampling has, to date, not yet demonstrated to improve overall survival according to a meta‐analysis [24]. Consequently, the disparity on examined lymph nodes during anatomical lung resection that is reported in these studies contributes to the confusion about intraoperative lymph node dissection. The reality of daily practice underlines this incongruence; from the Dutch national lung cancer audit, it can be deduced that in only a minority of patients, intraoperative lymph node sampling is performed according to guidelines [25].

In summary, the study of Guo et al. could partially aid in redefining the TNM classification and have an impact on adjuvant treatment modalities. This redefinition could be achieved by constructing less heterogeneous and more precise international guidelines on lymph node staging and by adhering and reporting according to these guidelines as well as possible. By doing so, this could also provide scientific opportunities, as research on this topic could be done in a more detailed and uniform manner.

Changing the TNM stage might be clinically relevant and could aid in future research endeavors. For the 9th edition of the TNM classification system, the International Association for the Study of Lung Cancer has proposed different concepts that should be investigated to change the N‐descriptor, like the aforementioned nodal zone concept and the number and exact location of lymph node metastases [11]. Integration of these concepts could be the path to more patient‐tailored treatment approaches and thus yield better survival results. Whether the N descriptor of the TNM classification will be changed or not, improved standardization of the current guidelines, and adherence to them, would be a good first step in the direction of improved nodal staging and patient‐tailored treatment modalities.

Because this is an invited editorial, no human or animal data were used, thus no ethical approvals or patient/animal consent applies to the draft of this manuscript.

## Conflicts of Interest

The authors are accountable for all aspects of the work in ensuring that questions related to the accuracy or integrity of any part of the work are appropriately investigated and resolved. The authors have no conflicts of interest to declare, and have all contributed to the draft of this manuscript.

## Data Availability

Data sharing not applicable to this article as no datasets were generated or analyzed during the current study.
